# Hepatocyte Models for Metabolic Dysfunction-Associated Steatotic Liver Disease: A Comparative Analysis of Non-HepG2 Cell Models

**DOI:** 10.3390/ijms27104453

**Published:** 2026-05-15

**Authors:** Anna Kotlyarova, Stanislav Kotlyarov

**Affiliations:** 1 Department of Pharmacy Management and Economics, Ryazan State Medical University, 390026 Ryazan, Russia; kaa.rz@yandex.ru; 2 Department of Nursing, Ryazan State Medical University, 390026 Ryazan, Russia

**Keywords:** metabolic dysfunction-associated steatotic liver disease, non-alcoholic fatty liver disease, hepatocytes, steatosis, lipotoxicity, cell models, HepaRG, Huh-7, immortalized human hepatocyte, primary human hepatocyte

## Abstract

Metabolic dysfunction-associated steatotic liver disease (MASLD) is a widespread condition with a complex pathogenesis. Cell-based models are important tools for studying the mechanisms underlying its development and progression. The aim of this review is to analyze the HepaRG, Huh-7, immortalized human hepatocyte (IHH), and primary human hepatocyte (PHH) cell lines for modeling and studying MASLD. HepaRG represents the most metabolically competent immortalized hepatocyte model with preserved biotransformation activity and a physiological bioenergetic response to lipid loading, making it valuable for pharmacological and toxicological studies. Huh-7 is distinguished by its accessibility and suitability for studying steatosis, lipotoxicity, insulin resistance, and paracrine mechanisms of fibrogenesis; however, its use is limited by its tumor origin, impaired carbohydrate metabolism, and low activity of xenobiotic-metabolizing enzymes. The IHH model occupies an intermediate position because of its non-tumor origin and is of interest for studies of senescence, epigenetic regulation, and signaling pathways involved in steatosis, although interpretation of results requires consideration of immortalization-related effects and specific metabolic limitations. PHH remains the most physiologically relevant platform for MASLD modeling, particularly in three-dimensional (3D) and microphysiological formats; however, its use is limited by high cost, interindividual variability, and the limited duration of the differentiated phenotype. Increasing model complexity—from two-dimensional (2D) monocultures to co-cultures, spheroids, and organ-on-chip systems—enhances physiological relevance and enables reproduction not only of steatosis but also of the inflammatory and fibrogenic components of MASLD progression, yet it reduces reproducibility and complicates standardization. Overall, none of the existing models is universal, and the optimal strategy is to select models according to the specific research question. A key direction for future research is the standardization of steatosis induction protocols and the unification of criteria for evaluating results.

## 1. Introduction

Metabolic dysfunction-associated steatotic liver disease (MASLD) is a widespread condition [1,2,3,4,5,6,7] closely associated with epidemiologically significant comorbidities such as obesity, metabolic syndrome, and diabetes mellitus [8,9,10,11,12,13,14,15,16,17,18]. The disease has a progressive course, beginning with simple steatosis—characterized by lipid accumulation in hepatocytes—which is eventually accompanied by inflammation and fibrosis (steatohepatitis); it may then progress to cirrhosis and increase the risk of developing hepatocellular carcinoma (HCC) [2,19,20]. The understanding of the pathogenesis of MASLD has evolved from a simple “two-hit” model to recognizing the disease as the result of the complex and long-term interactions between multiple factors, including dyslipidemia, insulin resistance, genetic predisposition, adipose tissue dysfunction, oxidative stress, and systemic inflammation [2,3,21,22,23,24,25]. This improved understanding has led to a revision of the nomenclature. In 2023, a new term, MASLD, was proposed to reflect the leading role of metabolic abnormalities [2,3,19,26]. It is important to note that this was not a simple replacement of one term with another; MASLD is a more comprehensive diagnosis than its predecessor, non-alcoholic fatty liver disease (NAFLD). MASLD is associated with increased cardiovascular risk [17,27,28]; therefore, it is not merely liver damage but a systemic metabolic disease.

Despite its widespread prevalence, the diagnosis and treatment of MASLD remain unresolved challenges [29,30,31,32]. In this regard, models of its various stages are necessary to better understand the mechanisms of the disease and to identify new therapeutic agents. This need is addressed through the use of animal models and cell-based experiments. Animals have long been used to model various diseases, including MASLD/NAFLD, and have served as the primary source of knowledge [33,34,35,36,37,38]. However, species-specific characteristics of animals must be taken into account. Rodents, which are most commonly used in laboratories, exhibit numerous phylogenetically determined differences from humans. In mice, for example, the main lipoproteins in the blood are high-density lipoproteins (HDLs), not low-density lipoproteins (LDLs) as in humans [39,40,41,42,43,44,45]. Ethical considerations also play a role, as reproducing a MASLD model may require a large number of laboratory animals; there are also time-related challenges, as determining the optimal modeling conditions can take a long time.

In this regard, there is growing interest in cellular models, which offer a number of advantages over animal models and are consistent with modern bioethical principles of replacement, reduction, and refinement (the 3Rs), which call for the replacement of animal models with alternative methods whenever possible [46,47,48,49,50]. Another advantage is the rapid generation of results, since the desired mechanism can be modeled much more quickly in cell lines, and it is easier to modify the modeling conditions by adjusting substance concentrations, exposure time, and other parameters. Diseases can be modeled in combination with other diseases by adding the necessary metabolites to the medium [51,52,53]. Cell lines do not raise the same ethical issues as animal models [46,47,48,49,50].

**Figure 1:**
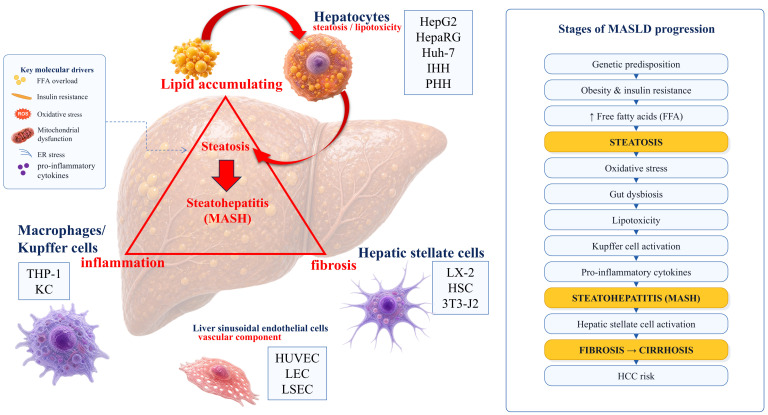
Mechanisms of MASLD pathogenesis and key cell lines used to model the disease and its pathogenetic components. Red arrows indicate the direction of MASLD progression and the transition from lipid accumulation and steatosis to inflammation, MASH, and fibrosis. Yellow blocks highlight the key pathological stages of MASLD progression, whereas light-blue blocks indicate contributing molecular and cellular mechanisms. Abbreviations: MASLD, metabolic dysfunction-associated steatotic liver disease; FFA, free fatty acid; MASH, metabolic dysfunction-associated steatohepatitis; HSC, hepatic stellate cells; LX-2, human hepatic stellate cell line LX-2; 3T3-J2, mouse embryonic fibroblast cell line 3T3-J2; HUVEC, human umbilical vein endothelial cell; LEC, liver endothelial cell; LSEC, liver sinusoidal endothelial cell; THP-1, human monocytic leukemia cell line THP-1; KC, Kupffer cell; ROS, reactive oxygen species; ER, endoplasmic reticulum.

Although cell lines also have a number of limitations, including the inability to replicate complex tissue architecture and intercellular interactions, cellular models have successfully established themselves in experimental research. Various cell lines are available for modeling different stages of MASLD pathogenesis (Figure 1). It is essential to distinguish between three categories of hepatocyte models: tumor cell lines, immortalized non-tumor hepatocytes, and primary human hepatocytes (PHHs), each of which has its own advantages and limitations. Tumor cell lines, such as HepG2, Huh-7, and HepaRG, offer high reproducibility, accessibility, and suitability for high-throughput experiments; however, they carry traces of tumor reprogramming, genomic instability, altered energy metabolism, a reduced or distorted profile of biotransformation enzymes, and disrupted lipid transport regulation. Immortalized human hepatocytes (IHHs) occupy an intermediate position. IHH cells do not originate from tumors, but their phenotype is modified by the immortalization process itself. PHHs, on the other hand, retain a metabolic profile closest to that of mature human hepatocytes; however, they remain a model with a limited shelf life, high donor variability, and rapid dedifferentiation in 2D culture. Therefore, these models are not interchangeable but should be considered based on their physiological relevance and experimental reproducibility. The HepG2 cell line is the primary focus of research interest [54,55,56,57,58,59,60,61,62,63,64,65] and was analyzed in detail in our previous review [66]. It has been shown that the selection of an optimal MASLD model using HepG2 must take into account the metabolic characteristics of these cells and align with specific research objectives. A variety of modeling techniques have been proposed, which have undergone a certain evolution: from simple 2D models to more complex 3D models and co-cultures.

Importantly, the existing cell lines allow for the modeling of only certain aspects of MASLD pathogenesis—primarily steatosis, lipotoxicity, and the associated intracellular signaling pathways [51,52,67]. They cannot reproduce systemic metabolic disorders (such as insulin resistance, dyslipidemia, and obesity), which are an integral part of the clinical diagnosis [8,18,24]. Nevertheless, such models remain an indispensable tool for studying the fundamental mechanisms of hepatocyte damage and for the initial screening of potential therapeutic compounds [51,53,68]. In this regard, various authors continue to use both the new (MASLD) and the old (NAFLD) terminology [19,53,69], since cellular models specifically assess the hepatic manifestations of the disease, which are better reflected by the older term. This review primarily uses the new terminology (MASLD), but to maintain scientific rigor when describing studies that specifically modeled NAFLD, that term is used.

The aim of this narrative review is to analyze the HepaRG, Huh-7, immortalized human hepatocyte (IHH), and primary human hepatocyte (PHH) cell lines for modeling and studying MASLD. The literature included in this review was identified through a comprehensive search of major biomedical databases, including PubMed and Scopus. The search strategy included combinations of keywords such as “cell models”, “MASLD”, “NAFLD”, “hepatocytes”, “HepaRG”, “Huh-7”, “IHH”, “PHH”, “steatosis”, and “lipotoxicity”. Particular attention was paid to experimental, translational, and clinical studies published after 2020 that investigated cellular models of NAFLD/MASLD. The reference lists of relevant articles were also analyzed to identify additional relevant studies and ensure the inclusion of key current data.

## 2. The HepaRG Cell Line for Modeling MASLD: Characteristics and Applications

The HepaRG cell line was derived in 2002 from a hepatocellular carcinoma associated with hepatitis C virus (HCV) infection. It is a bipotent hepatic progenitor cell line that differentiates into hepatocyte-like and cholangiocyte-like cells under suitable culture conditions. The donor of this cell line was a 35-year-old woman with chronic hepatitis C [70,71,72,73]. To acquire a primary hepatocyte-like phenotype, the cells must undergo differentiation (typically lasting 2 weeks in medium containing 1.7–2% dimethyl sulfoxide (DMSO) after reaching confluence) [72,74,75,76]. This results in the formation of a polarized epithelial layer with clear morphological and functional differentiation into islands of hepatocyte-like cells surrounded by cholangiocyte-like cells [72,74,77]. Differentiated HepaRG cells closely resemble normal hepatocytes. They exhibit high expression and activity of key cytochrome P450 (CYP450) biotransformation enzymes (notably CYP3A4 and CYP1A2), functional transporters, and polarity. They also secrete a wide range of hepatocyte-specific proteins, including albumin and apolipoprotein B (ApoB) and apolipoprotein E (ApoE) [76,77]. These properties make HepaRG the most physiologically relevant immortalized model for studying xenobiotic metabolism and the mechanisms of hepatotoxicity [72,74,77].

HepaRG is considered a functional substitute for PHHs for a number of applications [78]. Although the expression levels of some phase I enzymes (e.g., CYP2D6) and drug transporters in HepaRG cells may be slightly lower than in PHHs, this cell line possesses a number of undeniable practical advantages that make it an attractive alternative for many applications [70,79]. Unlike PHHs, which rapidly dedifferentiate in culture, HepaRG cells demonstrate significantly greater longevity, maintaining a stable phenotype for several weeks, which allows for long-term and repeated studies [70,79,80]. HepaRG cells are characterized by high viability and, importantly, express the α5β1 integrin, which provides better adhesion to the extracellular matrix compared to other cell lines [70]. Finally, unlimited availability and standardized culture protocols make working with HepaRG significantly simpler and more cost-effective compared to using donor PHHs, which is reflected in their wide availability as a cell model [79,80,81]. Despite their high functionality following differentiation, HepaRG cells are not identical to primary hepatocytes across all regulatory axes. Furthermore, the 2D model requires strict standardization of the protocol (timing, medium composition, and DMSO stage); otherwise, inter-batch variability increases, which must be taken into account when planning experiments and interpreting the obtained data [72,79,82].

A key difference between the HepaRG cell line and the more commonly used HepG2 cell line lies in the nature of their metabolic response to exposure to free fatty acids (FFAs) [83]. While in HepG2 cells, exposure to FFAs leads to suppression of mitochondrial respiration, which corresponds to the late stages of the pathological process, HepaRG cells demonstrate stimulation of both mitochondrial respiration (including basal, maximal, and adenosine triphosphate (ATP)-linked) and glycolysis [83]. This pattern of adaptive increase in energy metabolism in response to a lipid load is much closer to clinical data obtained from patients with simple steatosis and early stages of metabolic dysfunction-associated steatohepatitis (MASH) [83].

The current concept of MASLD emphasizes the metabolic basis of hepatic steatosis and its association with insulin resistance, lipotoxicity, mitochondrial dysfunction, and oxidative stress, which arise against a backdrop of disrupted lipid homeostasis [83]. Given the prevalence of HepG2 in 2D models, HepaRG is often considered a more physiologically accurate alternative in terms of metabolism for studies in which mitochondrial respiration and energy processes [83], as well as the proper functioning of detoxification systems and nuclear receptors and the reproduction of “early” adaptations in steatosis and the initial stages of steatohepatitis, are critical.

The approaches to modeling MASLD in HepaRG cells described in the literature [81,83,84,85,86,87,88,89] can be classified according to increasing levels of complexity and physiological relevance. Two-dimensional models remain the most common and basic. The fundamental difference between protocols using HepaRG and HepG2 is the need for prior differentiation and control of the cell population composition (the ratio of hepatocyte-like and cholangiocyte-like fractions). In 2D models using HepaRG, the most common inducers are those that mimic the key “parallel hits” of MASLD, namely FFAs (oleic acid (OA)/palmitic acid (PA)) in combination with bovine serum albumin (BSA) (often with an OA:PA ratio of 2:1) as the core axis of steatosis and lipotoxicity and hyperglycemia and/or hyperinsulinemia (in various combinations with FFAs) to establish a metabolic context resembling insulin resistance.

The simplest and most reproducible model suitable for screening is described in the work by Bitterer et al. [87]. Differentiated HepaRG cells were cultured for 24 h in serum-free medium and then incubated with a mixture of FFAs (PA and OA in a 1:2 ratio and a total concentration of 0.5 mM) conjugated with BSA. Treatment for 24 h resulted in significant accumulation of lipid droplets, confirmed by Oil Red O staining, which mimicked a key feature of steatosis [87]. The low total concentration of FFAs (0.5 mM) and the moderate exposure time (24 h) make this model suitable for simulating mild steatosis.

In the model by Ahn et al. [88], a short exposure to OA was used. To reproduce the pathogenic processes of MASLD in vitro, HepaRG cells were cultured with 10% fetal bovine serum (FBS), insulin (5 ng/mL), and hydrocortisone (50 μM). To induce steatosis, cells were treated with OA (0.2 mM) for 3 h [88]. The exposure time is extremely short (3 h), and the OA concentration (0.2 mM) is within the physiological range; however, the use of OA alone (without PA) limits the modeling of lipotoxicity.

An in vitro NASH model in the HepaRG cell line with extended induction is demonstrated in the work by Maseko et al. [83]. To induce steatosis, cells were treated for 24 h with a mixture of FFAs—OA and PA—in two regimens: at a 2:1 ratio (OA:PA, total concentration 1 mM) and a 1:1 ratio (total concentration 2 mM), previously conjugated with BSA at a molar ratio of FFA: BSA = 5.3:1, which approximates pathophysiological conditions, since in human plasma the normal molar ratio of FFA to albumin is approximately 0.5–2:1, and in metabolic syndrome it increases to 3–6:1. The main objective of the experiment was a comparative study of adaptations in cellular energy metabolism, specifically the assessment of changes in mitochondrial respiration, respiratory complex activity, triacylglyceride accumulation, and markers of lipotoxicity in HepaRG cells compared to the HepG2 cell line [83]. This model is the most methodologically sound 2D model for studying bioenergetic processes. Its two-mode design allows for the assessment of dose-dependent effects and the influence of the saturated fatty acid fraction.

The model of steatosis and the NAFLD-characteristic induction of CYP2E1 activity described in the study by Michaut et al. [89] is characterized by prolonged exposure and altered FFA composition. Differentiated HepaRG cells were cultured with insulin (5 μg/mL) and hydrocortisone. Steatosis was induced with stearic acid (SA) (100 μM) or OA (100 μM) for 7 days. A key condition was a 7-day incubation with 100 μM SA, which led to triglyceride (TG) accumulation and a significant increase in CYP2E1 activity, in contrast to OA [89]. This model differs fundamentally from standard models in its use of SA, which is a more saturated and lipotoxic analog of PA, and in the duration of exposure (7 days). CYP2E1 induction is a clinically significant marker associated with oxidative stress in MASLD. The model is specifically designed to study drug-induced hepatotoxicity in the context of steatosis. The key parameters of the described steatosis induction protocols are systematized in Table 1.

Microphysiological systems (MPSs) represent an intermediate level of complexity between classical 2D cultures and fully developed 3D models. The study by Wen et al. [85] describes a NAFLD model using HepaRG cells in an MPS based on a cycloolefin polymer (COP). A key technical advantage of COP over standard polydimethylsiloxane (PDMS) is the absence of adsorption of hydrophobic molecules (fatty acids and the fluorescent dye AdipoRed), which ensures precise control of inducer concentrations and reliable lipid detection. To induce steatosis and lipotoxicity, cells were treated for 24 h with the sodium salt of PA and the sodium salt of OA at a concentration of 300 μM each, individually or in a mixture (1:1 ratio), conjugated with a 30% solution of BSA [85]. The model assessed steatosis, apoptosis, the expression and secretion of functional hepatocyte markers, and CYP3A activity, confirming its suitability for screening lipotoxic effects and studying disease progression mechanisms. This study highlights an often-overlooked issue—the influence of culture medium on quantitative results when modeling steatosis. The adsorption of fatty acids by the walls of PDMS devices can lead to a systematic error in the assessment of the effective concentration of inducers and a distortion in the detection of accumulated lipids.

In their study, Bronsard et al. [86] described a method for modeling MASLD using three-dimensional organoids composed of HepaRG cells (80%), primary human macrophages (10%) (differentiated from peripheral blood mononuclear cells using the method described in [90]), and LX-2 stellate cells (10%)—so-called HML organoids. To induce steatosis and fibrosis characteristic of MASLD, the organoids were treated every other day from day 5 to day 14 of culture with a mixture of FFAs in a 1:2 ratio of SA (150 μM) to OA (300 μM), which were prepared with BSA. This protocol led to significant lipid accumulation (steatosis) and increased expression of pro-inflammatory and profibrogenic markers, as well as decreased CYP3A4 expression, which is relevant for modeling key pathophysiological features of the disease for subsequent assessment of drug toxicity [86]. The model reproduces three key aspects of MASLD pathophysiology: steatosis (HepaRG), inflammation (macrophages), and fibrogenesis (LX-2). The use of SA instead of PA distinguishes this protocol from most studies. Prolonged FFA treatment approximates the chronic nature of the disease.

HepaRG cells are used to create more complex bioengineered multicellular liver microtissues (BE-MLMs) formed from co-cultures of cell lines and primary cells. To form BE-MLMs, Cho et al. [81] used HepaRG, human umbilical vein endothelial cells (HUVECs), Kupffer cells (KCs), and hepatic stellate cells (HSCs) in a ratio of 61:13:13:13. Spheroids containing the four cell types were formed in microplates over 4 days, after which they were encapsulated in a hydrogel and cultured for 21 days in a medium containing a mixture of FFAs (0.33 mM PA and 0.66 mM OA with 1% BSA) and 10 ng/mL transforming growth factor-β1 (TGF-β1). This method sequentially models the key stages of NAFLD, namely FFA-induced steatosis in hepatocytes, subsequent inflammation due to the activation of KCs, and, finally, fibrosis through the activation of stellate cells and the deposition of collagen type I and fibronectin. The aim of the experiment was to reproduce the natural multicellular pathogenesis of the disease in vitro for the screening of therapeutic agents, in contrast to simplified models involving the direct administration of TGF-β1 [81].

Thus, the differentiated HepaRG cell line represents one of the most well-established platforms for modeling MASLD in vitro, occupying an intermediate position between easy-to-use but metabolically limited lines (HepG2) and physiologically relevant but expensive and variable PHHs. A key advantage of HepaRG is its ability to reproduce the pattern of mitochondrial respiration stimulation under FFA loading characteristic of early-stage MASLD, combined with a preserved biotransformation system (CYP450, constitutive androstane receptor (CAR), and pregnane X receptor (PXR)), making this line particularly valuable for pharmacological studies.

The progression of MASLD models based on the HepaRG cell line from 2D monolayers to multicellular 3D organoids demonstrates a sequential expansion of opportunities to study various aspects of MASLD pathogenesis. However, increasing model complexity is accompanied by a decrease in reproducibility, which necessitates a deliberate choice of complexity level in accordance with the specific research objective. A critical limitation of all the described models remains the lack of standardized protocols, specifically the variability in FFA composition, concentrations, exposure time, and HepaRG differentiation conditions, which hinders interlaboratory comparison of results. Developing a minimal set of mandatory protocol parameters and control endpoints appears to be a priority for enhancing the translational value of data obtained from these models.

Thus, the HepaRG cell line serves as a convenient platform for multi-level studies of various mechanisms and signaling pathways under conditions of steatosis and lipotoxicity. The MASLD model in HepaRG is suitable for analyzing neutral lipid accumulation, TG content, lipid class composition, regulators of lipogenesis and lipid transport (*SREBP1*, *FASN*, *ACC*, *SCD1*, *CD36*, etc.), as well as markers of β-oxidation and mitochondrial lipid catabolism (*PPARA*, *CPT1A*, etc.). Due to the high activity of its biotransformation systems and preserved regulation of the xenobiotic response, HepaRG is widely used to assess CYP induction or inhibition, to study CAR- and PXR-dependent detoxification pathways, and to test for hepatotoxicity and drug–drug interactions.

## 3. Characteristics and Applications of the Huh-7 Cell Line for Modeling MASLD

The Huh-7 cell line (Hepatoma, University of Health) was established in 1982 by Hidekazu Nakabayashi’s group from a highly differentiated HCC surgically removed from a 57-year-old man of Japanese descent [91,92,93,94]. Historically, Huh-7 and its derivative subclones (Huh7.5 and Huh7.5.1) have been recognized as the “gold standard” in virology, primarily for the study of HCV [92,95,96]. It was the use of Huh-7 in combination with the JFH-1 isolate that made it possible in 2005 to establish, for the first time, a reliable HCV infectious culture system capable of supporting the virus’s full life cycle, from entry into the cell to the production of infectious virions [95]. This property, along with the preservation of many hepatocyte functions, established Huh-7 as a key tool for studying viral replication mechanisms, screening antiviral drugs, and investigating interactions in the “virus–host” system [92,96]. However, in recent years, the cell line has been increasingly used to model metabolic liver diseases, including MASLD/NAFLD [84,97,98,99,100,101,102].

A key genetic characteristic of Huh-7 in the context of MASLD is its status as a homozygous carrier of the *PNPLA3* I148M variant (patatin-like phospholipase domain-containing protein 3) (rs738409) [103]. This polymorphism is one of the most extensively studied genetic risk factors for MASLD and MASH in humans and is associated with increased TG accumulation in the liver, impaired lipid droplet remodeling, and fibrosis progression [104,105,106,107]. Direct experiments on Huh-7 cells show that *PNPLA3* I148M expression (as opposed to the wild-type) induces TG accumulation [108]. The molecular mechanism involves impaired TG remodeling, increased protein localization on lipid droplets [108], and, as shown in other models, competitive inhibition of the activity of the major lipase adipose triglyceride lipase (ATGL) through sequestration of its common cofactor α/β-hydrolase domain-containing protein 5 [109]. Thus, the presence of *PNPLA3* I148M in a homozygous state may increase the baseline susceptibility of Huh-7 to steatosis and exacerbate the phenotype upon exposure to FFAs [103,108,109], which must be taken into account when using this model in MASLD studies.

Huh-7 cells are of tumor origin and share metabolic limitations similar to those of HepG2 cells, making them suitable for use as a model line for MASLD. In the Huh-7 cell line, the basal expression of key Phase I biotransformation enzymes (CYP450) and the nuclear receptors responsible for their regulation, such as the pregnane X receptor (PXR), are significantly reduced [92,110,111,112]. Under standard culture conditions, mRNA levels and activity, for example, of CYP3A4, account for only a small fraction (about 1.5%) of those in PHH [111,113], and the expression of the *NR1I2* (encoding PXR) and *NR1I3* (encoding CAR) genes is extremely low [92]. This indicates an attenuated xenobiotic response, which limits the use of native Huh-7 cells in drug metabolism studies, although protocols (such as prolonged culture and treatment with DMSO or cyclin-dependent kinase (CDK) inhibitors) exist to partially restore this function [110,111,112].

The Huh-7 cell line is characterized by limited secretion of very-low-density lipoprotein (VLDL) and ApoB [114]. This makes Huh-7 a convenient model for studying intracellular TG accumulation, as the “synthesis–export” balance is easily shifted toward the formation of lipid droplets upon the addition of excess fatty acids and sugars [115]. Unlike mature hepatocytes, Huh-7 cells do not express glucokinase (GCK) [116,117]. During the malignant transformation of hepatocytes, a switch in hexokinase isoenzymes occurs: the key enzyme of glucose sensitivity, GCK, is replaced by high-affinity hexokinase 2 (HK2) [117]. This leads to a disruption in the physiological regulation of metabolism in response to changes in glucose concentration. This limitation prevents the standard Huh-7 cell line from serving as an adequate model for studying glucose-dependent aspects of MASLD pathogenesis [116,117], making it more suitable for investigating lipotoxic stress and the effects of excess fatty acids. The Huh-7 cell line exhibits distinctive features of the insulin response, making it a physiologically relevant and widely used model for studying the mechanisms of insulin resistance (IR) [118,119,120]. Unlike the HepG2 cell line, Huh-7 cells do not exhibit overexpression of IGF-1 (insulin-like growth factor-1) [84,118]. Insulin signaling in Huh-7 is mediated by the classical phosphoinositide 3-kinase/protein kinase B (PI3K/Akt) cascade, and its disruption can be induced in vitro [118,120]. It is important to note that the insulin-induced IR model in Huh-7 cells allows for the study of the molecular mechanisms underlying impaired insulin sensitivity in the absence of pronounced cytotoxicity and mitochondrial dysfunction, which are characteristic of models using FFAs (e.g., PA) [120].

Working with the Huh-7 cell line requires strict standardization of culture protocols due to its sensitivity to environmental conditions, such as serum type and concentration, seeding density, and the use of albumin as a carrier for FFAs [94,121]. Variability in these parameters can significantly affect the metabolic phenotype of cells and, consequently, the reproducibility of results [121]. In addition, the cultivation of Huh-7 cells is associated with limitations regarding the duration of cultivation and the number of passages. This cell line is characterized by pronounced genetic instability and heterogeneity, including complex chromosomal rearrangements and massive loss of heterozygosity [94]. During prolonged cultivation and at high passage numbers, genetic and phenotypic drift occur, which can lead to changes in the functional characteristics of cells, including the activity of transporters and metabolic enzymes [92,122]. To minimize such changes and ensure model stability, it is recommended to use cells with low passage numbers [92,122].

Huh-7 cells efficiently accumulate lipids following treatment with FFAs and rapidly develop a steatotic phenotype after FFA treatment [97,99,100,123]. The Huh-7 cell line is suitable for co-cultures with stellate cells (LX-2) and for studying paracrine mechanisms, such as exosomal signaling pathways in fibrogenesis [102]. This extends the model from steatosis to the mechanisms of early MASH via hepatocyte signaling molecules.

The MASLD/NAFLD modeling protocols described in the literature using Huh-7 cells have been systematized and are presented in Table 2 in order of increasing complexity and physiological relevance.

2D models using the Huh-7 cell line represent the most common and technically straightforward approach. The protocols described vary significantly in terms of the composition of the inducers, allowing us to identify two fundamental strategies for inducing steatosis: FFA-induced steatosis (the classical model) [97,99,100,123] and carbohydrate induction using fructose or glucose [124].

In the study by Khamphaya et al., to model steatosis in vitro, Huh-7 cells were treated with 500 μM PA in a BSA vehicle for 24 h, which led to lipid accumulation confirmed by Oil Red O staining [99]. In the study by Takahara et al., Huh-7 cells and primary rat hepatocytes were used to model steatosis and lipoapoptosis in vitro. To induce steatosis, cells were treated with a mixture of PA and OA at concentrations ranging from 200 to 800 μM in a 1% BSA vehicle for 24 h and, to model apoptosis, with 800 μM PA for 8–24 h [100]. In the study by Chávez-Tapia et al., Huh-7 cells were exposed for 24 h to a mixture of PA and OA in a molar ratio of 1:2 (PA:OA) at concentrations ranging from 100 to 1200 μM (100, 200, 400, 600, and 1200 μM), previously conjugated with BSA in a 4:1 molar ratio of FFAs to BSA, to model lipotoxicity. In this model, steatosis (accumulation of lipid droplets, assessed by Nile Red staining and flow cytometry), an inflammatory response, oxidative stress, and apoptosis were induced [123].

In the study by Zhou et al., Huh-7 cells were cultured for 24 h with a mixture of PA and OA in a 1:2 molar ratio at a total concentration of 1200 μM, conjugated with 300 μM fat-free BSA, to model steatosis and mitochondrial dysfunction in steatosis [97].

The in vitro method described in the study by Lee et al. [102] uses two cell lines, Huh-7 and LX-2. In this study, Huh-7 serves as a model, while the LX-2 hepatic stellate cell line is used to assess fibrogenic activation. Huh-7 cells were incubated with PA (0.4 mM) for 8 h to model lipid overload and trigger a pathological response. Exosomes were isolated from the supernatant of the treated hepatocytes and then added to LX-2 cells at concentrations of 50 or 100 μg/mL. Disease progression from steatosis to fibrosis (the NASH stage) was observed. In this context, the study investigated how signals from steatotic hepatocytes (via exosomes enriched with specific microRNAs (miRNAs), such as miR-192) activate stellate cells, leading to increased expression of fibrogenic markers (α-smooth muscle actin (α-SMA), TGF-β, and collagen type I alpha 1 chain (COL1A1)) [102].

A separate approach is described in the study by Windemuller et al., in which the Huh-7 cell line was cultured in DMEM medium containing 10% FBS and exposed for 24 h to iso-osmotic (400 mOsm/L) media containing either glucose alone (0.65–0.72 mmol/L) or a constant total monosaccharide concentration of 0.72 mmol/L with an increasing fructose:glucose (F:G) molar ratio ranging from 0.58 to 0.67 to study the effect of sugar composition on lipogenesis. It was found that an increase in the molar ratio of fructose to glucose enhances the synthesis of triglycerides and cholesterol and that fructose promotes lipogenesis in hepatocytes [124]. This model reproduces an alternative axis of MASLD pathogenesis—de novo lipogenesis stimulated by fructose—without the direct action of exogenous FFAs. This complements FFA-induced models and allows for the separation of the contributions of substrate (carbohydrate) overload and direct FFA lipotoxicity to the development of steatosis.

An analysis of the methods suggests that most 2D protocols using Huh-7 cells employ FFA concentrations in the range of 400–1200 μM with a 24 h exposure. The upper values (1200 μM) are supraphysiological and may induce nonspecific cytotoxicity, thereby masking pathogenetically significant mechanisms. Protocols with a dose-dependent design [100,123] are the most informative, allowing for the differentiation of adaptive and damaging responses. It is important to note that the protocols by Khamphaya et al. [99] and Lee et al. [102] use PA without OA. Monocomponent PA is more lipotoxic than the PA + OA mixture, since OA has a partially protective (anti-apoptotic) effect by directing PA toward esterification. These models specifically simulate maximal lipotoxicity rather than the typical metabolic profile of MASLD, where a mixture of FFAs is present.

The co-culture of Huh-7 cells with the LX-2 hepatic stellate cell line represents an intermediate level of complexity between an isolated 2D model and 3D microtissues. Two main approaches are described in the literature: direct co-culture [101] and indirect co-culture via exosomal transfer [102]. Direct co-culture, for example, is described in the work of Barbero-Becerra et al. [101], where the Huh-7 cell line and the LX-2 hepatic stellate cell line were used to model cellular interactions in steatosis. To induce lipid accumulation, cells were treated with a mixture of FFAs (OA and PA in a 2:1 ratio, total concentration 1200 μM) in a BSA vehicle for 24 h [101]. Indirect co-culture via exosomal transmission was described above using the example of the work by Lee et al. [102]. The indirect exosomal model has a fundamental advantage—it allows for the isolation and identification of specific molecular mediators of intercellular communication. Direct co-culture replicates the combination of paracrine, juxtacrine, and exosomal mechanisms but does not allow the contribution of each mechanism to be isolated. Both models extend the 2D system from simple steatosis to the mechanisms of early MASH.

Asadollahi et al. [98] described the most complex 3D model based on Huh-7—liver microtissues (LMTs) consisting of Huh-7, THP-1, LX-2, and HUVEC cell lines in a 7:2:2:1 ratio, encapsulated in a composite hydrogel. The model was induced by treating the LMT with a mixture of FFAs—OA (6.6 × 10^−4^ M) and PA (3.3 × 10^−4^ M) in a 2:1 ratio, conjugated with 1% fat-free BSA [98]. This model includes four cellular components that replicate key parenchymal and non-parenchymal liver populations: hepatocytes, represented by Huh-7 (70%), macrophages/KCs (THP-1 (20%)), stellate cells (LX-2 (20%)), and endothelial cells (HUVECs (10%)). Encapsulation in a liver-derived extracellular matrix (ECM)-based hydrogel provides a 3D architecture, intercellular contacts, and a biomechanical environment more closely resembling in vivo conditions. The four-cell composition allows modeling not only steatosis but also the inflammatory (THP-1) and fibrogenic (LX-2) components of MASLD progression to the early stages of MASH/fibrosis. A comparative analysis of Huh-7 cell line models of varying complexity is provided in Table 3.

The concentrations of FFAs used in various protocols vary by a factor of 12—from 100 μM to 1200 μM [97,123]. This creates a fundamental problem for the comparability of results, since cellular responses to 200 μM and 1200 μM FFAs differ qualitatively (from adaptive steatosis at low FFA doses to necrosis and apoptosis at high doses). High doses (1200 μM) are used to obtain a visually pronounced, easily detectable phenotype, but they model acute toxicity rather than the pathophysiology of NAFLD.

Thus, the Huh-7 cell line occupies a distinct niche in the arsenal of in vitro MASLD/NAFLD models, combining practical advantages with unique biological characteristics. Huh-7 is optimal as a mechanistic and screening step for comprehensive modeling of steatosis, inflammation, and apoptosis in 2D models [123], for studying mitochondrial dysfunction and metabolic reprogramming [97], for investigating the mechanisms of the transition from steatosis to fibrosis [102], for reproducing the complete pathogenic cascade of NAFLD in 3D models [98], and for studying the role of fructose in de novo lipogenesis [124]. At the same time, the interpretation of data obtained from Huh-7 requires constant consideration of the line’s metabolic limitations. The development of co-culture and 3D models based on Huh-7 demonstrates the potential for constructing biologically complex systems that reproduce the full pathogenic cascade of MASLD. A stepwise strategy (starting with 2D screening, followed by co-culture, and concluding with 3D validation) appears to be the most rational approach to integrating Huh-7 into research programs.

## 4. Characterization and Application of the IHH (Immortalized Human Hepatocyte) Cell Line for Modeling MASLD

Immortalized human hepatocytes are derived from non-neoplastic liver tissue of an adult donor (healthy tissue obtained during surgical intervention) and immortalized via stable transfection with simian virus 40 large T antigen (SV40 large T antigen; SV40T) [125]. The fundamental difference between IHHs and the most common hepatocellular cell lines (HepG2, Huh-7, and HepaRG) lies in their non-neoplastic origin, which conceptually enhances confidence in the results of modeling metabolic pathology. However, this advantage does not make IHHs equivalent to PHHs or a fully functional in vivo liver model. Immortalization using the SV40 large T antigen alters key cellular regulatory pathways, including p53- and pRb-dependent cell cycle control, stress responses, replicative aging, and genomic stability. Therefore, IHHs should be viewed not as a replacement for PHHs but as a reproducible intermediate model useful for analyzing specific intracellular mechanisms of steatosis, senescence, epigenetic regulation, etc. [125,126,127,128,129,130,131].

IHHs retain several key characteristics of differentiated hepatocytes [125]. Thanks to these properties, IHHs have been used as a model for studying lipid metabolism and steatosis [132], as well as for studying the molecular mechanisms and pathways of fibrogenesis [133]. Due to their ability to form multicellular spheroids, this line is also used to study intercellular interactions underlying fibrogenesis and tissue remodeling [134]. However, when interpreting data, particularly regarding stress responses and peroxisomal metabolism, it is important to note that the IHH cell line has well-established genetic defects, such as the absence of functional PEX7 protein (peroxisomal biogenesis factor 7). This leads to impaired import of proteins containing a peroxisomal targeting signal type 2 (PTS2), plasmalogen deficiency, and, consequently, potential disruptions in lipid homeostasis and antioxidant defense mechanisms [128]. Although a direct link between the PEX7 mutation and classical oxidative stress pathways in IHHs has not been established, it is known that peroxisomal dysfunction in hepatocytes can secondarily induce mitochondrial stress and disrupt redox homeostasis [135].

IHHs occupy a unique intermediate niche between PHHs and tumor cell lines, representing a balance between physiological relevance and reproducibility. PHHs are considered the most physiologically relevant platform for studying liver physiology and pathology, as well as for the preclinical evaluation of drug metabolism and toxicity [136,137,138]. However, the widespread use of PHHs in basic research and high-throughput screening tests is significantly limited by a number of factors, such as the low availability of high-quality donor material, dependence on surgical resections and organs unsuitable for transplantation, the high cost of isolation and cultivation, and marked inter-donor variability, which hinders the standardization and reproducibility of results [139,140,141]. On the other hand, tumor cell lines (HepG2 and Huh-7) exhibit high reproducibility and scalability but carry significant metabolic shifts due to their tumor origin. IHHs, having been immortalized from normal (non-tumor) tissue, combine moderate biological relevance with the reproducibility of an immortalized system.

The IHH cell line is widely used as an in vitro model for studying various aspects of liver metabolism, including glucose, lipid, and lipoprotein metabolism [127,128,142,143], as well as in more recent studies of signaling pathways [133] and epigenetic mechanisms [129]. Using IHH cells, it has been shown that key pathological processes, such as steatosis [129], hyperinsulinemia-induced aging [144], and oxidative stress [145], are associated with alterations in fatty acid metabolism, reactive oxygen species (ROS) production, and mitochondrial enzyme function, making this cell line suitable for screening compounds that potentially affect hepatocellular mitochondrial function.

Among the main limitations of using IHH cell lines are the effects associated with the use of viral oncogenes, particularly the SV40 large T antigen. The mechanism of immortalization is based on the effect of the SV40 T antigen on key cell cycle regulators—the p53 and retinoblastoma (pRb) proteins—which leads to the inactivation of the corresponding signaling pathways and allows cells to bypass replicative senescence and proliferate indefinitely [126,146,147,148,149]. Such intervention may disrupt normal cellular responses, causing genomic instability such as polyploidy and structural chromosomal abnormalities, and significantly alter the transcriptional profile of cells compared to the original primary cultures [126,150,151]. At the same time, IHH cells represent a promising platform for modeling MASLD in vitro, although this approach does not overcome the fundamental limitations associated with the absence of a fully developed mature hepatocyte phenotype. Compared to PHHs, IHHs offer greater accessibility, cultivation stability, and experimental reproducibility; however, they are inferior to PHHs in terms of physiological relevance, maturity of the metabolic program, and the ability to directly model donor-specific characteristics. Consequently, IHH is best suited as a model for screening individual mechanisms and comparative data validation, but not as an equivalent to PHH. Furthermore, the widespread use of IHH requires further investigation, including the expression of key metabolic enzymes, as well as genomic instability.

Recent studies have utilized IHH as a platform to analyze how acetyl-CoA-dependent processes are linked to changes in gene regulation and epigenetic alterations in steatosis. Furthermore, IHH cells are actively used to study cellular aging (senescence) in the context of MASLD/MASH—an area explored in two independent studies (Baboota et al. [130], Bonnet et al. [152]), which defines a specific niche for this cell line that is not characteristic of HepG2, Huh-7, or HepaRG.

The protocols described are organized in order of increasing complexity and physiological relevance. 2D models using IHH demonstrate significant heterogeneity in steatosis induction protocols. Analysis of the studies described below reveals three strategies that differ in the composition of inducers, concentrations, and experimental objectives, namely, induction with single-component OA (the most common and dominant method); induction with a mixture of FFAs in various ratios; and an alternative metabolic induction (insulin and microbial metabolites). The corresponding protocols are summarized in Table 4.

An analysis of the published protocols reveals a fundamental difference between IHH models and those based on HepG2 and Huh-7 cells. Most of the studies described use OA as the sole FFA inducer of steatosis [129,130,152,153,154,155], whereas for HepG2/Huh-7, a PA + OA mixture (usually in a 1:2 ratio) is the standard. This difference has profound implications for the interpretation of results, since OA predominantly induces “mild” steatosis—the accumulation of triglycerides without pronounced apoptosis or ER stress—whereas PA is the primary mediator of lipotoxicity. Thus, IHH models primarily reproduce early steatosis and subacute lipid overload, rather than the full spectrum of lipotoxic damage characteristic of MASH. Two distinct clusters can be identified: a low-dose cluster (OA 50 μM with 48 h exposure) [153,154,155] and a medium-dose cluster (OA 200–300 μM) with variable exposure ranging from 24 h [130,152] to 4 days [129]. The protocol by Nagarajan et al. [156], featuring a dose-dependent design (OA 150/300/450 μM, 24 h), occupies an intermediate position and allows for the construction of a dose–response curve.

Two studies use fundamentally different inducers. L’homme et al. [157] used stearate (C18:0, 250 μM, 16 h) in combination with insulin and dexamethasone to model acute lipotoxic stress, using OA as a control. This design creates a contrast between saturated and unsaturated FAs, rather than a standard comparison of FFA treatment versus control. Deslande et al. [158] used a combination of insulin (100 nM) with hippurate (500 μM), a microbial metabolite—without exogenous FFA—which reflects the growing interest in the microbiome–liver axis in the pathogenesis of MASLD and represents a unique approach [158].

In the protocol by Cansby et al. [131], a high dose of PA (400 μM) is added at a minimal OA concentration (50 μM), creating a PA:OA ratio of 8:1. This differs radically from the physiological PA:OA ratio in plasma (1:1.5–1:2) and creates a maximally lipotoxic environment, with the aim of inducing ER stress to study germinal center kinase III (GCKIII kinase).

IHH is used to model MASLD for the purpose of studying signaling kinases of the STE20 family [131,153,154,155]. All four studies share a common focus on the role of the GCKIII kinase subfamily (MST3, MST4, STK25, TAOK3, and MAP4K4) in the regulation of hepatocyte lipid homeostasis and lipid droplet dynamics. Three of them use an identical protocol: in the studies by Xia et al. [153], Caputo et al. [154], and Anand et al. [155], the IHH cell line was treated with 50 μM OA for 48 h, leading to lipid accumulation.

The second area of research is cellular senescence in the context of NAFLD and NASH [130,152]. In the study by Bonnet et al., the human hepatocyte lines HepG2 and IHH were used to model the role of cellular senescence in the pathogenesis of NASH. First, senescence was induced, and to enhance steatosis, 200 µM OA was added for the IHH cell line and 400 µM OA for the HepG2 cell line, leading to lipid accumulation confirmed by Oil Red O staining and TG measurement, as well as metabolic changes [152]. In the study by Baboota et al., IHH and LX-2 cell lines were used to model cellular senescence and steatosis in vitro. Steatosis was induced by treating senescent cells with 200 µM OA for 24 h. To investigate the anti-inflammatory and anti-fibrotic effects of bone morphogenetic protein 4 and its antagonist Gremlin 1 under conditions mimicking NASH, three-dimensional spheroids derived from IHH and LX-2 (in a 24:1 ratio) treated with TGF-β1 (5 ng/mL) for 48 h were used [130]. Interestingly, TGF-β1 is used as the inducer in the spheroids rather than FFA, indicating the modeling of the late inflammatory-fibrogenic stage of NASH rather than initial steatosis. Second, the IHH:LX-2 ratio of 24:1 differs significantly from other co-culture models. For example, Bronsard et al. [86] use a HepaRG:macrophages:LX-2 ratio of 8:1:1, while Cho et al. [81] use a HepaRG:HUVEC:KC:HSC ratio of 61:13:13:13. A higher ratio of hepatocytes to stellate cells may limit the severity of the fibrogenic component. Both studies [130,152] used a two-step protocol—first inducing senescence, then adding OA (200 μM) to enhance steatosis. This approach defines a specific niche for IHH, as senescence models on HepG2/Huh-7 are significantly less common. It is noteworthy that Bonnet et al. [152] observed differences in sensitivity among cell lines; for example, to induce comparable steatosis in senescent cells, 200 μM OA was required for IHH and 400 μM for HepG2, indicating that IHH is more sensitive to OA.

A third area is epigenomics and acetyl-CoA-dependent processes [129]. In the study by Assante et al. and other similar studies, to model steatosis, IHH cells were treated with 300 μM OA conjugated to albumin for 4 days, which led to significant lipid accumulation and an increase in the level of the DNA damage marker γH2AX, detected by immunofluorescence [129]. The chronic model (300 μM OA, 4 days) revealed a link between steatosis, changes in acetyl-CoA metabolism, epigenetic modifications, and DNA damage. This protocol with prolonged exposure most closely approximates chronic steatosis in vivo.

A fourth approach involves modeling MASH-level lipotoxic stress [157]. In their study, L’homme et al. used the IHH cell line cultured with 20 μM insulin and 50 nM dexamethasone to model the lipotoxicity of hepatocytes characteristic of MASH. The phenotype was induced by a 16 h treatment with 250 μM stearate (C18:0) to create lipotoxic stress, using OA (C18:1) at the same concentration as a control, to study the regulation of GDF15 expression in response to stress [157]. The use of stearate instead of PA, as well as the inclusion of insulin and dexamethasone in the culture medium, creates a more physiological hormonal context and addresses specific stress responses.

A fifth approach—the microbiome–liver axis [158]—represents a fundamentally new type of model without exogenous FFA. In their study, Deslande et al. used IHH to model insulin resistance and steatosis in vitro, subjecting the cells to a 24 h co-treatment with 100 nM insulin and 500 μM hippurate to assess hippurate’s ability to attenuate insulin-induced lipid accumulation. Key validation methods included quantitative staining of intracellular triglycerides and glucose uptake analysis [158].

The sixth area of research involves the comparative validation of cellular models [156]. In their study, Nagarajan et al. used various cell lines, including IHHs, to model steatosis in vitro. Lipid accumulation was induced by 24 h treatment with either OA (0, 150, 300, or 450 μM) or an FFA mixture (PA/OA/linoleate in a 1:2:1 ratio) to assess the ability of various cellular models to accumulate triglycerides in response to a lipid load [156]. This study is an example of a dose-dependent design using a three-component FFA mixture that includes linoleic acid (LA) (PA:OA:LA = 1:2:1). LA is a polyunsaturated fatty acid and triggers a different mechanism of lipotoxicity via lipid peroxidation, distinct from that of PA or OA.

The IHH cell line occupies a unique niche among in vitro MASLD/NAFLD models due to its non-tumorigenic origin combined with the reproducibility of an immortalized system. Its intermediate position between PHHs and tumor cell lines makes IHH particularly attractive for applications where metabolic fidelity is critical. At the same time, it maintains the ability to serially compare conditions and doses. 2D models based on IHH monolayer cultures primarily reproduce early steatosis and subacute lipotoxicity, while the 2D format ensures high reproducibility and compatibility with high-throughput screening (HTS). In contrast, the IHH + LX-2 3D spheroids model the inflammatory-fibrogenic stage of MASH. The presence of stellate cells enables the reproduction of intercellular interactions; however, steatosis itself has not been described as an endpoint for this model. The reproducibility of the 3D model is rated as moderate, and its compatibility with HTS is rated as limited due to the complexity of spheroid formation and handling.

A critical limitation remains the significantly smaller volume of validation data for IHH compared with HepG2, Huh-7, and HepaRG. Standardization of the basic protocol and systematic comparison with other cell lines under identical conditions appear to be promising tasks for elevating the status of IHH as a standardized platform for modeling MASLD.

## 5. Characterization and Application of PHH for Modeling MASLD

Primary human hepatocytes (PHHs) are not a cell line in the strict sense but rather a primary population of mature parenchymal liver cells isolated from donor tissue (resection specimens, organs unsuitable for transplantation) and used in fresh or cryopreserved form [67]. Unlike all tumor and immortalized cell lines discussed in previous sections (HepG2, Huh-7, HepaRG, and IHH), PHHs retain a complete tissue-specific transcriptional and epigenetic program of a mature hepatocyte. A key element of this program is the expression of a set of liver-enriched transcription factors, primarily the HNF family (hepatocyte nuclear factor (HNF) family, including hepatocyte nuclear factor 4α (HNF4α)) [159]. It is this unique transcriptional network that ensures a high level of metabolic and detoxification functions, including the synthesis of albumin and urea, as well as the activity of phase I and II enzymes. Experimental comparisons show that tumor lines, such as HepG2, and immortalized hepatocytes (e.g., HepaFH3) exhibit significantly reduced levels of these key markers of mature hepatocytes [160], and HepaRG cells show marked differences in the expression of metabolic genes compared with PHHs [161].

The primary value of PHHs for MASLD modeling lies in preserving human metabolic integrity. This includes unaltered lipid metabolism, namely FFA uptake, de novo lipogenesis, mitochondrial and peroxisomal β-oxidation, esterification, lipid droplet formation, and lipid export as part of ApoB-containing lipoproteins. The latter is particularly important, as a defect in VLDL/ApoB secretion is one of the key limitations of tumor cell lines, disrupting the balance of the TG synthesis–export process [67,68,162,163]. In addition, PHHs maintain physiological carbohydrate metabolism with insulin-dependent switching between lipogenesis and oxidation, as well as fully functional xenobiotic metabolism (CYP450 isoenzymes and transporters), which is critical for pharmacological and toxicological studies [68,163,164,165].

Even the most metabolically competent of the cell lines—HepaRG (after differentiation)—generally falls short of PHHs in terms of certain CYP450 profiles, although it may surpass them in certain activities [70,79]. The HepG2 and Huh-7 tumor cell lines are characterized by low or partial expression of CYP450 enzymes, as described in previous sections.

A unique advantage of PHHs is the ability to study the effects of human risk alleles. In PHH 3D spheroids, it has been shown that the *TM6SF2 E167K* variant is associated with increased intracellular fat due to reduced secretion of ApoB-containing particles, as well as with differences in the expression of cholesterol, fatty acid, and carbohydrate metabolism pathways [166]. The *PNPLA3* I148M genetic variant is capable of significantly altering lipid metabolism in human hepatocytes, inducing TG accumulation and changes in lipid composition [167,168]. A key consequence of these metabolic changes is the activation of programmed cell death pathways. Specifically, in PHHs carrying the I148M variant, the development of endoplasmic reticulum (ER) stress, mitochondrial dysfunction, and, ultimately, cell death via ferroptosis has been demonstrated [167]. These data directly link genetic risk to specific cellular mechanisms of liver damage progression. This circumstance underscores the critical importance of using PHHs in preclinical studies. Unlike tumor cell lines with a fixed and often abnormal karyotype, PHHs retain donor-specific genetic variants, including *PNPLA3* I148M, which is essential for modeling MASLD, where genetic predisposition is one of the key factors in interindividual variability in disease progression and response to therapy [167].

One of the key advantages of PHHs is the ability to directly account for donor sex when modeling MASLD. This is particularly significant because sex hormones not only systemically influence adipose tissue distribution and insulin resistance but also directly modulate hepatocellular lipid metabolism. In an experimental model of steatosis in PHHs derived from male and female donors, FFA loading induced comparable intracellular accumulation of triacylglycerides; however, female PHHs demonstrated a higher capacity for VLDL secretion, whereas male PHHs were characterized by a more pronounced transcriptional response of lipid metabolism genes. Furthermore, 17β-estradiol reduced triacylglyceride accumulation predominantly in female PHHs, whereas the effects of testosterone were sex-specific [169].

Thus, the baseline condition and reactivity of the cells may be influenced by the donor’s age, sex, body mass index, presence of diabetes or dyslipidemia, prior treatment, the degree of steatosis in the donor liver, the isolation method, cryopreservation and recovery after thawing, and other factors. Consequently, different donor PHH preparations may vary in baseline lipid content, β-oxidation activity, capacity for ApoB/HDL secretion, sensitivity to PA/OA loading, inflammatory cytokine profiles, and drug responses. Therefore, the use of PHHs requires mandatory specification of donor characteristics, the number of donors, viability after thawing, duration of exposure, composition, and the FFA:BSA molar ratio, as well as a set of functional control endpoints. To improve reproducibility, it is desirable to include at least several independent donors or to specify that the results reflect the response of a specific donor preparation.

At the same time, the designation of PHHs as the “gold standard” requires a fundamental clarification. In the context of MASLD modeling, PHHs should be viewed not as a universal platform but as a reference system for reproducing the functions of mature human hepatocytes. The advantages and the aforementioned properties of PHHs are most fully realized only when using freshly isolated or high-quality cryopreserved cells and under strictly controlled culture conditions. In classical 2D monolayer culture, PHHs rapidly lose their differentiated phenotype, which is accompanied by a decrease in albumin and urea synthesis, a change in polarity, a reduction in CYP enzyme activity, and a restructuring of the transcriptional and proteomic profiles [137,170,171]. Therefore, results obtained in 2D PHH models, especially during prolonged exposure to free fatty acids, should be interpreted with consideration of the possible contribution of dedifferentiation, rather than solely steatogenic or lipotoxic effects.

Overall, modeling MASLD using PHHs has a number of other significant limitations. The limitations of PHHs can be divided into practical and biological categories. Practical limitations include limited availability, high cost, ethical challenges, and the logistical difficulties involved in obtaining high-quality material [139,165]. Biological limitations primarily include inter-donor variability [138,170,172], which is both a drawback (a reproducibility issue, since different donors may exhibit different baseline lipid levels and reactivity to steatogenic stimuli) and an advantage, as it enables the study of personalized aspects of MASLD; for example, in 3D spheroids, a reproducible inter-donor difference in the degree of steatosis induction under identical conditions has been demonstrated [138,165,173].

Conventional 2D monolayer PHH culture rapidly loses its differentiated phenotype (dedifferentiation), which limits long-term experiments [165,171]. Sandwich cultures and spheroids extend the functional lifespan of cells to up to 3 weeks [174], while 3D spheroid culture ensures the preservation of viability and metabolic functions for at least 5 weeks [138]. However, despite these advances and the introduction of perfusion systems, existing in vitro models are generally limited to a time window of 2 to 4 weeks [138,165,170,174]. This period remains incomparable to the actual years of MASLD development and its progression to MASH in humans, underscoring the need for further refinement of models for studying chronic pathologies.

Thus, when designing experiments using PHHs, it is necessary to distinguish among three levels of physiological relevance. The first level is the classical 2D monoculture, which is primarily suitable for short-term analysis of steatosis, acute stress responses, and preliminary toxicity assessments but is limited by rapid dedifferentiation. The second level consists of sandwich cultures and micropatterned co-cultures, which better preserve polarity, transport functions, and the activity of biotransformation systems, a factor particularly important for pharmacokinetic and toxicological studies. The third level consists of 3D spheroids, multicellular MPSs, and organ-on-chip platforms, which allow for extended experimental study times and partially replicate intercellular interactions, as well as the inflammatory and fibrogenic components of MASH. However, as physiological complexity increases, throughput, cost-effectiveness, and ease of standardization decrease. Therefore, PHHs should be used as a reference model in a cascade strategy, rather than as the sole mandatory standard for all types of MASLD research.

The described MASLD/NAFLD modeling protocols using PHHs are distinguished by an exceptional variety of formats—ranging from the classic 2D monolayer to perfused multicellular MPS. Below is a systematic overview of all the described protocols, presented in a summary table (Table 5), followed by a detailed analytical breakdown by level of complexity.

Classic 2D models of PHHs are represented by several protocols [175,176,177,178], which share a short exposure time (24 h) and the use of PA and/or OA as inducers. This approach is the simplest and fastest, but its limitations include the rapid dedifferentiation of PHH in monolayer culture.

In the study by Sharma et al., to induce a lipid load, PHH cells were treated separately with each of the presented FFAs: saturated (PA), cis-unsaturated (OA), and trans-unsaturated (elaidic acid, EA), as well as with their combination, at a concentration of 200 μM each for 24 h. It should be noted that cells treated with a mixture of all three FFA types failed to survive after 24 h, indicating synergistic toxicity of the combination that significantly exceeded the effect of each component. After steatosis induction, the glucagon-like peptide-1 (GLP-1) receptor agonist exendin-4 (10 nM) was added to the model to assess its effect on lipid accumulation, apoptosis, the unfolded protein response (UPR), and autophagy. The aim of the study was to model a key feature of NAFLD—hepatocellular steatosis—and to investigate the mechanisms by which GLP-1 agonists reduce lipid load through the modulation of ER stress and the induction of macroautophagy [175]. This protocol is distinguished by its unique use of three types of FFAs, both separately and in combination.

In the study by Wanninger et al., to induce lipid accumulation, PHH cells were treated with PA (0.3 mM) or OA (0.6 mM) for 24 h; this was confirmed by Oil Red O staining and led to increased TGF-β secretion [176]. In the study by Kirovski et al., to model steatosis in vitro, PHHs were treated with PA at concentrations of 0.4, 0.6, and 0.8 mM for 24 h, which was confirmed by quantitative TG analysis, and the induction of RANTES chemokine expression was studied to assess the effect of steatosis on the chemokine response [177]. The protocols by Wanninger et al. and Kirovski et al. use single-component FFA, which allows for the differentiation of specific responses to PA and OA. Mahli et al. supplemented the model with an assessment of the pro-inflammatory response alongside steatosis. In their study, Mahli et al. used PHH and the HepG2 cell line to model steatosis and associated inflammation, incubating them with 0.4 mM OA in the presence of BSA for 24 h. Lipid accumulation was assessed using Oil Red O staining and TG quantification, while the pro-inflammatory response was evaluated via quantitative reverse transcription polymerase chain reaction (qRT-PCR) of markers interleukin 8 (IL-8), intercellular adhesion molecule 1 (ICAM-1), and chemokine (C-C motif) ligand 5 (CCL5) and Western blotting of nuclear factor κB (NF-κB) activation [178].

In the study by Breher-Esch et al., PHH, Huh-7 and HepG2 cell lines were used to model the steatosis characteristic of NAFLD. The phenotype was induced by treatment with a mixture of PA and OA in a 1:1 ratio at a total concentration of 0.5 mM for 72 h or 7 days, with additional simulation of inflammation by adding tumor necrosis factor alpha (TNF-α) (5 ng/mL). The aim of the experiment was to create a model for screening drug therapy targets, and lipid accumulation and steatosis were confirmed by quantitative Oil Red O staining, followed by transcriptomic analysis [179].

Notably, all 2D protocols on PHH cells use FFA concentrations in the range of 200–800 μM, which corresponds to physiological and borderline ranges, avoiding supraphysiological doses (>1 mM) characteristic of several protocols on HepG2/Huh-7 cells.

The sandwich format represents an intermediate level of complexity, allowing for a significant extension of the functional lifespan of PHH compared to a 2D monolayer. The most complex protocol in this group is that of Murphy et al. [180]. It consists of a PHH sandwich culture (SCHH) with exposure to OA and PA in a non-standard 1:2 ratio (total concentration 0.5 mM) together with the pro-inflammatory cytokines TNF-α (1 ng/mL) and interleukin 6 (IL-6) (1.2 ng/mL) for 72 h. This model is unique in that it specifically reproduces not only steatosis but also the clinically observed reduction in the expression and function of drug-metabolizing enzymes (CYP450 and UDP-glucuronosyltransferases (UGTs)) and membrane transporters (organic anion-transporting polypeptides (OATPs), sodium taurocholate cotransporting polypeptide (NTCP), bile salt export pump (BSEP), and multidrug resistance-associated proteins (MRPs)), making it indispensable for the preclinical assessment of the impact of MASLD on pharmacokinetics. Notably, Huh-7 was used in this study only as a screening model during the preliminary optimization phase, while the final experiments were conducted on PHHs [180].

In their study, Kwon et al. proposed a protocol for modeling MASLD using cryopreserved PHHs from multiple donors [173]. Key elements of the protocol included prolonged (7-day) cell culture in a 3D collagen sandwich format in the presence of a mixture of FFAs. In this model, the researchers were able to study the development of steatosis, insulin resistance, mitochondrial dysfunction, and the inflammatory response, as well as changes in the expression of key genes [173]. As proof-of-concept, the authors demonstrated that the use of the acetyl-CoA carboxylase inhibitor firsocostat (GS-0976) effectively reduced lipid accumulation and manifestations of insulin resistance under the simulated conditions [173]. These results confirm that the proposed platform is suitable for preclinical screening and for studying the mechanisms of action of potential drug candidates for the treatment of MASLD.

In another study, steatosis was induced in micropatterned co-cultures (MPCCs), commercially known as HEPATOPAC, which is a bioengineered platform in which PHHs are organized into microislands surrounded by supporting stromal cells (3T3-J2). After the MPCC culture had stabilized, the cells were cultured in a DMEM-based medium containing insulin–transferrin–selenium supplement (final insulin concentration 1.1 μM), 200 nM L-glutamine, 0.7 ng/mL glucagon, 10 nM dexamethasone, and 10% FBS, as well as either a “high glucose + fructose” variant (10 g/L glucose and 1 g/L fructose) or a combination of these conditions. The results demonstrate the suitability of MPCC for both testing metabolic drugs and assessing drug-induced steatosis [181].

The conditioned medium model represents an indirect co-culture. In their study, Piras et al. used PHHs and the LX-2 cell line. The hepatocytes were treated with 1 mM PA (in combination with 1% BSA), 10 mM fructose, or their combination for 48 h, after which LX-2 cells were incubated with the conditioned medium to assess profibrotic transcriptional responses [182]. The PA + fructose combination mimics the synergistic effect characteristic of the Western diet; however, the PA concentration (1 mM) is supraphysiological.

PHH 3D spheroids represent the best-characterized model system for long-term modeling of MASLD. Key advantages include an extended functional lifespan of up to several weeks, the preservation of polarity and intercellular contacts, and the ability to sustain prolonged (7–21 days) exposure to FFAs while maintaining viability and albumin secretion.

In their study, Prill et al. used 3D spheroids derived from PHHs, cultured in Williams E medium supplemented with insulin, transferrin, selenite, dexamethasone, and 10% FBS, to model steatosis and investigate the effects of the *TM6SF2* E167K genetic variant. To induce lipid accumulation, the spheroids were treated with a mixture of OA and PA in a 2:1 ratio (total concentration 160 or 320 μM) in combination with BSA for up to 10 days. TG content was assessed, lipid droplets were visualized by Nile Red staining, and ApoB secretion was analyzed by Western blotting [166].

In their study, Kozyra et al. used three-dimensional spheroids derived from PHH to model steatosis and insulin resistance. To induce steatosis, the spheroids were cultured in serum-free Williams E medium for 7–14 days, treated with a mixture of palmitic and oleic acids (PA:OA in a 1:1 ratio, final concentration up to 320 μM), and conjugated with BSA to ensure bioavailability. In an alternative protocol, a combination of high concentrations of monosaccharides (glucose and fructose) with insulin (0.1 nM to 10 nM) was used to activate de novo lipogenesis. The development of steatosis was confirmed by staining lipids with Nile Red dye, and the development of insulin resistance was assessed by increased expression of gluconeogenesis genes (*PCK1* (phosphoenolpyruvate carboxykinase 1), and *G6Pase* (glucose-6-phosphatase)) and the *PDK4* marker, as well as by decreased GSK3β phosphorylation in response to insulin stimulation [183].

The protocols by Prill et al. [166] and Kozyra et al. [183] are examples of a standardized approach using OA + PA mixtures at concentrations of up to 320 μM. In the study by Kozyra et al., concentrations of insulin (0.1–10 nM) and monosaccharides (glucose and fructose) are also varied, creating a “metabolic syndrome” model, which represents the most physiologically comprehensive approach among spheroid protocols. For this protocol, a dose-dependent steatosis (160–320 μM) was demonstrated, peaking by days 14–21, along with the induction of lipogenesis and, critically for pharmacology, the maintenance of CYP3A4 protein levels despite steatosis.

In the study by Bell et al. PHH spheroids (3D PHH spheroid system) cultured in serum-free medium were used to model steatosis, a key component of NAFLD. The modeling was performed by treating the spheroids with cyclosporin A at a concentration of 30 µM for 48 h, which led to a significant accumulation of neutral lipids, confirmed by LipidTOX Green staining. This expands the scope of application of PHH spheroids beyond the classical NAFLD model and demonstrates their suitability for screening drug hepatotoxicity [138].

The transition from PHH monoculture to multicellular systems is necessary to reproduce the full pathogenic cascade of MASH, including inflammatory and fibrogenic components. The protocols described represent a continuum from co-cultured spheroids to complex MPS and organ-on-chip platforms.

In their study, Ströbel et al. [184] used three-dimensional spheroids derived from a co-culture of PHH, KC, LEC and HSC to model NASH. The NASH phenotype was induced over 10 days by adding FFAs (167 μM), high concentrations of glucose and fructose (a total of 22.5 mmol/L), and LPS (5 μg/mL). Key disease features were modeled: steatosis (TG accumulation, Nile Red staining), inflammation (secretion of IL-6, TNF-α, and chemokines), and fibrosis (secretion of procollagen I, Sirius Red staining) [184].

A series of studies by Kostrzewski et al. demonstrates the evolution of the MPS platform [185,186,187]. The first protocol (LiverChip^®^) utilized a PHH monoculture with a high dose of FFAs. To model steatosis, a key feature of NAFLD, PHHs were cultured in a three-dimensional perfusion system (LiverChip^®^) in a medium containing 2 nM insulin and 5.5 mM glucose. Steatosis was induced over 7–14 days by adding a mixture of OA and PA in a 2:1 ratio (total concentration 600 μM), pre-conjugated with BSA, to the medium, which led to significant lipid accumulation. Modeling of the early stage of the disease was confirmed by Oil Red O staining, measurement of FFA uptake from the medium, and detection of reduced metabolic activity, specifically a decrease in the activity of cytochromes CYP3A4 and CYP2C9 [185].

The second protocol expanded the system to a three-cell co-culture. To model NASH, a three-dimensional co-culture of PHHs, KCs, and human hepatic stellate cells was used in a perfused three-dimensional MPS, cultured in a medium containing a mixture of saturated and unsaturated FFAs, physiological concentrations of insulin, and sugars. The NASH phenotype was induced over a period of at least 2 weeks, with inflammation further enhanced by the addition of LPS (0.5 ng/mL, starting on day 8), leading to the development of steatosis, inflammation (secretion of IL-6 and TNF-α), and fibrosis (secretion of procollagen 1, tissue inhibitor of metalloproteinases-1 (TIMP-1), and fibronectin) [186].

The third protocol was designed to enhance the fibrogenic component by adding TGF-β, additional LPS, fructose, and cholesterol. To model fibrosis, a co-culture of PHHs, KCs, and human hepatic stellate cells was used in an MPS. The cells were cultured for 14 days in a medium containing a mixture of FFAs and physiological concentrations of insulin and glucose. To enhance the fibrotic phenotype, LPS (1 ng/mL) (lipopolysaccharides from *Escherichia coli*), TGF-β (1 ng/mL), fructose (500 μM), and cholesterol (50 μg/mL) were additionally applied. This combination led to significant deposition of collagen I and expression of α-SMA, as well as secretion of profibrotic markers (TIMP-1 and procollagen 1). This made it possible to model and quantitatively assess fibrosis as a key feature of progressive NASH [187].

The study by Hellen et al. describes an MPS characterized by the longest exposure time among the protocols described (up to 19 days). The authors used a unique composition of free fatty acids—a three-component mixture of PA:OA:LA in a ratio of ≈33:50:28 (total concentration 100 μM)—as well as pathological concentrations of insulin (800 nM) and glucose (11 mM). For the control condition, physiological conditions were used: 200 nM insulin, 5.5 mM glucose, and 20 μM FFA. The models were confirmed by measuring insulin resistance, intracellular TG accumulation, and the secretion of pro-inflammatory chemokines [188]. This design is the only one among those described in which both pathological and control concentrations of all medium components are clearly defined.

Feaver et al. described a microfluidic model featuring a co-culture of PHHs, HSCs, and macrophages in a collagen sandwich. The lipotoxic phenotype was induced over 10 days using a medium containing 25 mM glucose, 6900 pM insulin, and a mixture of FFAs (65 μM OA and 45 μM PA). Insulin concentrations of 690 pM and glucose concentrations of 5.6 mM were used as physiological controls. Thus, this protocol simulates a 10-fold increase in insulin levels, which corresponds to severe hyperinsulinemia. The aim of the experiment was to create a translational model for studying disease mechanisms and screening therapeutic agents [189].

In the study by Freag et al., the “NASH-on-a-chip” MPS was used to model NASH, in which PHHs, KCs, stellate cells, and sinusoidal endothelial cells were co-cultured in a three-dimensional collagen gel under dynamic perfusion. The NASH phenotype was induced over 10 days with a mixture of OA (0.66 mM) and PA (0.33 mM) acids, as well as LPS (10 μg/mL). The model reproduces hepatocyte ballooning (a histological hallmark of NASH), stellate cell activation, and the secretion of pro-inflammatory (monocyte chemoattractant protein 1, macrophage inflammatory protein 1α, and TNF-α) and profibrotic (COL1A1 and TIMP-1) markers [190].

The models described form a clear gradient ranging from simple 2D systems (hours) to complex multicellular platforms (weeks), in which each level expands the range of reproducible aspects of pathogenesis but reduces throughput and increases cost. 2D monolayers [175,176,177,178] enable rapid (24 h) modeling of steatosis and initial stress responses but are limited by dedifferentiation and the absence of intercellular interactions. Sandwich cultures and MPCCs [173,180,181] extend functional lifespan to 3–7 days, allowing for the assessment of subacute effects, including downregulation of CYP450/transporters and a complex transcriptomic response. The addition of cytokines (TNF-α and IL-6) extends the model to include an inflammatory component. 3D PHH spheroids [138,166,183] provide the longest duration (up to 21 days) and the greatest physiological relevance among monoculture formats. The preservation of CYP450 activity, the dose-dependence of steatosis (160–320 μM), and the ability to study genetic variants make this format optimal for mechanistic studies and comparative genetics. Multicellular 3D models and MPS [184,185,186,187,188,189,190] reproduce the full pathogenic cascade of NASH, including steatosis, inflammation, HSC activation, and profibrotic markers. The addition of LPS enhances the inflammatory component, while perfusion ensures the delivery of nutrients and the removal of metabolites, approximating conditions to sinusoidal blood flow. Pharmacological validation confirms the translational value. However, these models are less reproducible, significantly more expensive, and extremely difficult to scale for HTS.

PHH are considered the “gold standard” among in vitro cellular models of MASLD due to their comprehensive metabolic profile, which includes intact lipid and carbohydrate metabolism, complete xenobiotic metabolism (CYP isoenzymes, transporters), and donor-specific genetic risk variants. None of the tumor (HepG2 and Huh-7) or immortalized (HepaRG, IHH) cell lines fully replicates this complex set of characteristics.

The evolution of culture formats—from 2D monolayers through sandwich cultures and 3D spheroids to multicellular MPS and organ-on-chip platforms—consistently expands the range of reproducible aspects of pathogenesis. Pharmacological validation using therapeutic candidates confirms the translational value of PHH platforms. At the same time, none of the existing PHH models overcomes a fundamental limitation: the temporal compression of a multi-year process. Although 3D culture and perfusion techniques have extended the duration of experiments to 7–10 days, MASLD/MASH is a chronic disease that develops over many years. The use of acute, high-stress conditions (high doses of FFAs, LPS, and cytokines) may shift the underlying mechanisms toward acute lipotoxicity. Reproducing mature fibrosis with full architectural remodeling remains beyond the capabilities of existing models. Furthermore, the absence of inter-organ interactions (adipose tissue–liver crosstalk, contribution of the microbiota) limits systemic relevance.

## 6. Comparison of Models and Prospects for Future Research

**Figure 2:**
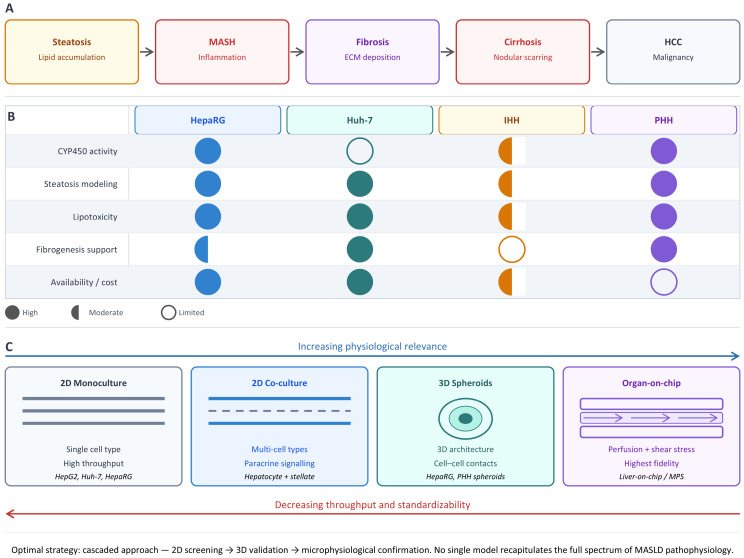
Comparative analysis of in vitro MASLD cell models. Comments: (**A**) Stages of MASLD progression: from simple steatosis, defined as the accumulation of neutral lipids in hepatocytes, through metabolic dysfunction-associated steatohepatitis (MASH; inflammation) and fibrosis to cirrhosis, characterized by nodular scarring, and hepatocellular carcinoma (HCC; malignant transformation). (**B**) A matrix comparing the suitability of cellular models (HepaRG, Huh-7, IHH, and PHH) based on five parameters is presented: cytochrome P450 (CYP450) activity, reproducibility of steatosis, lipotoxicity modeling, support for fibrogenesis, and practical availability of the model. The size of the circle reflects the relative magnitude of the parameter: ○ (empty circle)—the parameter is absent or poorly supported; ◐ (half-filled circle)—moderate magnitude; ● (fully filled circle)—high magnitude. The assessment is qualitative and based on a synthesis of the literature reviewed in this overview; it does not constitute a formal quantitative meta-analysis. For the “availability/cost” parameter, ● indicates higher experimental availability and lower cost, whereas ○ reflects limited availability, high cost, or complex logistics for obtaining the material. (**C**) Spectrum of experimental complexity of culture systems: 2D monoculture (single cell type, high-throughput compatibility); 2D co-culture (multiple cell types and paracrine signaling); 3D spheroids (three-dimensional architecture, cell–cell interactions); organ-on-a-chip system (perfusion, highest physiological relevance). Increasing system complexity is accompanied by reduced reproducibility and standardizability. An optimal research strategy involves a stepwise approach: 2D screening → 3D validation → microphysiological confirmation. Abbreviations: IHH—immortalized human hepatocytes; PHH—primary human hepatocytes; CYP450—cytochrome P450; MPS—microphysiological systems.

Thus, the high demand for studying the pathogenesis of MASLD has contributed to the widespread use of various cellular models. A comparative analysis of cell lines used to model MASLD demonstrates the absence of a universal model suitable for addressing all research questions. Each of the systems reviewed (HepaRG, Huh-7, IHH, and PHH) occupies a specific niche in the spectrum of in vitro studies, possessing a unique combination of physiological relevance, experimental accessibility, and reproducibility (Figure 2).

The HepaRG cell line represents a balanced compromise between the ease of use of immortalized cell lines and metabolic competence. Its key advantage lies in the preservation of an active biotransformation system (CYP450) and the ability to reproduce the adaptive pattern of mitochondrial respiration in response to a lipid load, characteristic of the early stages of steatosis in humans. The increasing complexity of HepaRG-based models (from 2D monocultures to multicellular organoids) allows for a sequential expansion of the range of parameters studied, from isolated steatosis to inflammation and fibrogenesis.

The Huh-7 cell line is an optimal tool for studying lipotoxicity, mechanisms of insulin resistance, and the “steatosis–fibrosis” transition in paracrine systems (e.g., during co-culture with LX-2 cells). The presence of the homozygous *PNPLA3 I148M* variant makes it a genetically relevant model for a significant portion of the population. However, the use of Huh-7 requires strict control of the experimental design, since high concentrations of free fatty acids (e.g., 900–1000 μM linoleate) can cause acute cytotoxicity [191], whereas at other fatty acid ratios, concentrations up to 1200 μM are used to model the early stages of MASLD [123,192]. Furthermore, the low activity of phase I biotransformation enzymes, particularly CYP3A4 (whose expression and activity under standard Huh-7 culture conditions are minimal compared to primary human hepatocytes [92,110,111]) limits the use of this cell line in pharmacokinetic studies without prior cell preconditioning [111,112].

The IHH cell line occupies a unique intermediate position due to its non-tumor origin. This makes it valuable for verifying results obtained in cancer cell lines, particularly in the study of cellular aging (senescence) and epigenetic regulation in steatosis. However, the presence of genetic defects associated with the immortalization method (in particular, peroxisome dysfunction due to a *PEX7* mutation) requires caution when interpreting data concerning lipid homeostasis and antioxidant defense.

PHH should be considered the most physiologically relevant reference model of mature human hepatocytes for key MASLD modeling tasks. Their unique value lies in the preservation of human lipid and carbohydrate metabolism, CYP isoenzyme and transporter activity, secretion of ApoB-containing lipoproteins, and donor-specific genetic risk variants. However, the application of PHH is limited by rapid dedifferentiation in 2D culture, inter-donor variability, high cost, limited availability, and the inability to fully replicate systemic MASLD. Therefore, PHHs are most suitable as a reference and validation platform, particularly in 3D, sandwich, and microphysiological formats, whereas for primary screening and standardized mechanistic experiments, it is more rational to use other reproducible cell lines.

It is important to note that traditional models based on cell lines, particularly in the form of two-dimensional monocultures, have not yet been proven to be an ideal solution for modeling MASLD and have a number of significant drawbacks [193,194,195]. In particular, such systems cannot reproduce the complex tissue architecture and intercellular interactions between hepatocytes, macrophages (Kupffer cells), stellate cells, and the endothelium, which play a key role in the progression of the disease from simple steatosis to MASH and fibrosis [193,194]. Moreover, even when co-cultures are used, cell lines often do not fully reflect the functional characteristics of the corresponding primary cells, and many physiological signals from the microenvironment remain unaccounted for [195]. Cell models may differ significantly from real cells in terms of metabolism, as we discussed in our previous review, which included a discussion of the HepG2 cell line [66].

Thus, the prospects for future research in the field of MASLD modeling are linked not so much to the search for an “ideal” model as to the development of multi-level research strategies. A rational approach would be to combine different pathways to model specific aspects of MASLD pathogenesis, taking into account their metabolic characteristics. Protocol standardization is also a key task. The current variation in inducer concentrations, FFA ratios, exposure times, and differentiation conditions makes it difficult to compare results across laboratories. The development of minimum reporting standards for in vitro MASLD models could significantly enhance the translational value of the data obtained and accelerate the process of developing effective therapies for this socially significant disease.

A separate area of standardization should focus on accounting for other clinical factors, such as including sex as a mandatory variable in the description of in vitro MASLD models. Clinical and experimental data show that MASLD exhibits marked sex dimorphism. In men, the prevalence of the disease is generally higher in young and middle age, whereas in women, the risk of steatosis, MASH, and fibrosis increases after menopause, which is associated with a reduction in the protective effects of estrogen on fat distribution, insulin sensitivity, mitochondrial function, oxidative stress, and inflammatory responses in the liver. Large population-based studies have also shown that age, body mass index, and metabolic factors modify the risk of MAFLD/MASLD differently in men and women, requiring consideration of sex when interpreting both clinical and experimental data [196,197,198,199].

For cell lines, the sex of the original donor should be specified, and it should be noted that tumor origin, aneuploidy, immortalization, and other characteristics of the cell line used may distort natural sex differences. This is particularly important because cell lines originate from a specific donor of a specific sex but carry tumor—or immortalization-related alterations that may mask natural sex differences in metabolism. For example, HepaRG was derived from a woman and Huh-7 from a man, whereas for PHHs, the donor’s sex can be experimentally determined and therefore represents an important design parameter.

When describing MASLD models, it is advisable to specify the donor’s sex or the line’s origin, as well as the composition of all culture medium components: insulin, glucocorticoids, and added sex hormones, since these factors can alter lipid accumulation, VLDL/ApoB secretion, the expression of lipid metabolism genes, and the cellular response to FFA loading. Recent data obtained in PHHs indicate that cells of male and female origin may respond differently to estrogen, progesterone, and testosterone under conditions of experimental steatosis [169]. In this regard, for PHHs and other donor-dependent systems, stratification by sex should be performed whenever possible.

Special attention is required when modeling conditions associated with significant hormonal changes, including menopause and pregnancy. Pregnancy is accompanied by profound changes in hepatic immunology and regulation by chorionic gonadotropin, estrogens, progesterone, prolactin, melatonin, prostaglandins, and other hormonal factors [200]. Standard models of steatosis in hepatocytes cannot automatically be considered relevant for studying MASLD in a gestational context without specific protocol adaptation.

Thus, cellular models remain an indispensable tool for studying the fundamental mechanisms of hepatocyte damage and for the initial screening of potential therapeutic compounds [51,53,68]. The selection of a particular cell line for modeling MASLD or its individual components must take into account the characteristics of these cells and correspond to the specific research objectives.

## 7. Conclusions

A comparative analysis of cell lines used to model MASLD demonstrates that there is no single universal system capable of reproducing all aspects of the disease’s pathogenesis. Each of the lines reviewed has known advantages and limitations. HepaRG is characterized by its availability and metabolic competence, making it well-suited for modeling early steatosis and biotransformation. Huh-7 can be effectively used to study lipotoxicity, insulin resistance, and fibrogenesis; however, it requires monitoring for cytotoxicity and is not suitable for pharmacokinetic studies without prior modification. IHH is a valuable validation model due to its non-tumor origin, but genetic defects from immortalization limit its use in lipid metabolism studies. PHH is considered the “gold standard” because it has the appropriate metabolic profile; however, its high cost, inter-donor variability, and limited culture lifespan restrict its application.

Thus, various cell lines are used to model MASLD, and their selection depends on the ultimate goal of the study. Given the pathogenetic complexity of the disease, both models that study individual links in the pathogenesis and more complex systems that reproduce various cell–cell crosstalk interactions are of interest.

## Figures and Tables

**Table 1:** Steatosis inducers: systematization of protocols.

Type of FFA	FFA, Composition, Concentrations	Time	References
OA + PA	OA:PA 2:1, total 1 mM; OA:PA 1:1, total 2 mM	24 h	[83]
PA + OA	PA:OA 1:2, total 0.5 mM	24 h	[87]
OA	OA 0.2 mM	3 h	[88]
SA or OA (separately)	SA 0.1 mM; OA 0.1 mM	7 days	[89]
PA + OA	PA:OA 1:1, total 0.3 mM	24 h	[85]
SA + OA	SA + OA 1:2; total 0.45 mM	9 days	[86]
PA + OA	PA + OA 1:2; total 0.99 mM	14–21 days	[81]

Abbreviations: FFA, free fatty acid; OA, oleic acid; PA, palmitic acid; SA, stearic acid.

**Table 2:** Steatosis induction protocols on the Huh-7 cell line.

Type of FFA	Concentrations of FFA	Time	References
PA	0.5 mM	24 h	[99]
PA + OA; PA	PA + OA, 0.2–0.8 mM; PA 0.8 mM	24 h (steatosis); 8–24 h (apoptosis) 24 h	[100]
PA + OA	PA:OA 1:2; 0.1–1.2 mM	24 h	[123]
PA + OA	PA:OA 1:2; 1.2 mM	24 h	[97]
PA	PA 0.4 mM	8 h	[102]

Abbreviations: FFA, free fatty acid; OA, oleic acid; PA, palmitic acid.

**Table 3:** Comparative characteristics of Huh-7 cell line MASLD models of varying complexity.

Feature	2D (Huh-7)	Co-Culture (Huh-7 + LX-2)	3D (Huh-7 + THP-1 + LX-2 + HUVEC)
Steatosis	Yes	Yes	Yes
Inflammation	Partial (intracellular)	No (without macrophages)	Yes (THP-1)
Fibrosis	No	Yes (via LX-2)	Yes (LX-2 in 3D)
Paracrine mechanisms	Exosomes (indirect)	Exosomes + direct contacts	Multicellular
Endothelium	No	No	Yes (HUVEC)
MASLD stage	Steatosis, lipotoxicity	Steatosis → early fibrosis	Steatosis → MASH → early fibrosis
High-throughput screening (HTS)	High	Medium	Low
Reproducibility	High (with protocol standardization)	Medium	Lower (complex assembly)
Translational value	Screening	Mechanistic	Highest among those described

**Table 4:** Systematization of steatosis induction protocols in the IHH cell line.

Type of FFA	Concentrations	Time	References
OA	0.05 mM	48 h	[153]
OA	0.05 mM	48 h	[154]
OA	0.05 mM	48 h	[155]
OA	0.2 mM	24 h	[130]
OA	0.2 mM	24 h	[152]
OA	0.3 mM	4 days	[129]
OA alone or PA:OA:LA, 1:2:1	0.15 mM, 0.3 mM, 0.45 mM;	24 h	[156]
OA + PA	OA 0.05 + PA 0.4 mM	48 h	[131]
SA control: OA	0.25 mM	16 h	[157]
Insulin	100 nM	24 h	[158]

Abbreviations: FFA, free fatty acid; OA, oleic acid; PA, palmitic acid; LA, linoleic acid; SA, stearic acid.

**Table 5:** Examples of MASLD modeling protocols in PHH-based systems.

Format	Inductor	Concentration	Time	Cell Composition	References
2D	PA, OA, EA	0.2 mM each one	24 h	PHH	[175]
2D	PA or OA	PA 0.3 mM; OA 0.6 mM	24 h	PHH	[176]
2D	PA	0.4 mM, 0.6 mM, 0.8 mM	24 h	PHH	[177]
2D	OA	0.4 mM	24 h	PHH (+ HepG2)	[178]
2D (PHH, Huh-7, HepG2)	PA:OA 1:1 ± TNF-α (tumor necrosis factor alpha)	0.5 mM FFA + 5 ng/mL TNF-α	72 h/ 7 days	PHH (Huh-7, HepG2)	[179]
Sandwich (SCHH)	OA:PA 1:2 + TNF-α + IL-6	0.5 mM FFA + 1 ng/mL TNF-α + 1.2 ng/mL IL-6	72 h	PHH	[180]
3D collagen sandwich system	PA:OA 1:5	0.025 mM +0.125 mM,	7 days	PHH	[173]
Micropatterned co-cultures (MPCCs)	Sodium palmitate: sodium oleate 2:1 high concentrations of glucose and fructose (HGF)	0.5 mM FFA HGF: 10 g/L glucose, 1.0 g/L fructose	2–7 days	PHH; PHH + 3T3-J2 (mouse embryonic fibroblast cell line 3T3-J2)	[181]
PHH → post-treatment environment → LX-2	PA PA + fructose	PA 1 mM; fructose 10 mM	48 h	PHH → LX-2	[182]
3D spheroids	OA:PA 2:1	0.16 mM: 0.32 mM	up to 10 days	PHH	[166]
3D spheroids	Two independent approaches: 1. FFA: a 1:1 mixture of PA and OA. 2. Monosaccharides + insulin: glucose + fructose + insulin	1. FFA: 0.32 mM For monosaccharides: 11 mM glucose + 10 mM fructose combined with 1720 nM insulin. (in experiments with FFA, the concentration of insulin varied from 0.1 nM to 10 nM, but its effect on the degree of steatosis was minimal)	7–14 days	PHH	[183]
3D spheroids	Cyclosporine A	0.03 mM	48 h	PHH	[138]
3D spheroids co-culture	FFA + glucose + fructose + lipopolysaccharide (LPS)	0.167 mM of FFA; 22.5 mM of monosaccharides; 5 µg/mL of LPS	10 days	PHH + KC + LEC + HSC	[184]
3D (LiverChip)	OA:PA 2:1	0.6 mM	7–14 days	PHH	[185]
3D MPS	FFA + insulin + sugars + LPS	HEP-FAT medium containing a mixture of FFA in physiological concentrations + LPS 0.5 ng/mL	≥14 days	PHH + KC + HSC	[186]
3D MPS	FFA + LPS + TGF-β + fructose + cholesterol	LPS 1 ng/mL; TGF-β 1 ng/mL; 0.5 mM fructose; 50 µg/mL cholesterol	14 days	PHH + KC + HSC	[187]
3D MPS	FFA (PA:OA:LA ≈33:50:28) + insulin + glucose	0.1 mM of FFA; 800 pM insulin; 11 mM glucose	up to 19 days	PHH	[188]
Collagen sandwich	FFA (OA:PA 13:9) + insulin + glucose	0.11 mM of FFA (OA 0.065 mM + PA 0.045 mM); 25 mM glucose; 6900 pM insulin	10 days	PHH + HSC + macrophages	[189]
Organ-on-chip (NASH-on-a-chip)	OA + PA + LPS	~1 mM FFA (OA 0.66 mM + PA 0.33 mM) + LPS 10 µg/mL	10 days	PHH + KC + HSC + LSEC	[190]

Abbreviations: 2D, two-dimensional; 3D, three-dimensional; EA, elaidic acid; FFA, free fatty acid; HGF, high glucose and fructose; HSC, hepatic stellate cell; IL-6, interleukin-6; KC, Kupffer cell; LA, linoleic acid; LEC, liver endothelial cell; LPS, lipopolysaccharide; LSEC, liver sinusoidal endothelial cell; LX-2, human hepatic stellate cell line LX-2; MPS, microphysiological system; MPCCs, micropatterned co-cultures; NASH, non-alcoholic steatohepatitis; OA, oleic acid; PA, palmitic acid; PHH, primary human hepatocyte; SCHH, sandwich-cultured human hepatocyte; TGF-β, transforming growth factor beta; TNF-α, tumor necrosis factor alpha.

## Data Availability

No new data were created or analyzed in this study. Data sharing is not applicable to this article.
